# Involvement of Hypoxia-Inducible Factor 1-α in Experimental Testicular Ischemia and Reperfusion: Effects of Polydeoxyribonucleotide and Selenium

**DOI:** 10.3390/ijms232113144

**Published:** 2022-10-29

**Authors:** Pietro Antonuccio, Giovanni Pallio, Herbert Ryan Marini, Natasha Irrera, Carmelo Romeo, Domenico Puzzolo, Jose Freni, Giuseppe Santoro, Igor Pirrotta, Francesco Squadrito, Letteria Minutoli, Antonio Micali

**Affiliations:** 1 Department of Human Adult and Childhood Pathology, University of Messina, 98125 Messina, Italy; pietro.antonuccio@unime.it (P.A.); romeo.carmelo@unime.it (C.R.); amicali@unime.it (A.M.); 2 Department of Clinical and Experimental Medicine, University of Messina, 98125 Messina, Italy; gpallio@unime.it (G.P.); hrmarini@unime.it (H.R.M.); nirrera@unime.it (N.I.); ipirrotta@unime.it (I.P.); fsquadrito@unime.it (F.S.); 3 Department of Biomedical, Dental Sciences and Morphofunctional Imaging, University of Messina, 98125 Messina, Italy; puzzolo@unime.it (D.P.); jose.freni@unime.it (J.F.); giuseppe.santoro@unime.it (G.S.)

**Keywords:** testis, ischemia-reperfusion, rat, selenium, polydeoxyribonucleotide, hypoxia-inducible factor 1-α, phosphorylated extracellular signal-regulated kinases 1/2, germinal epithelium, Leydig cells

## Abstract

Polydeoxyribonucleotide (PDRN) is an agonist of the A2A adenosine receptor derived from salmon trout sperm. Selenium (Se) is a trace element normally present in the diet. We aimed to investigate the long-term role of PDRN and Se, alone or in association, after ischemia-reperfusion (I/R) in rats. The animals underwent 1 h testicular ischemia followed by 30 days of reperfusion or a sham I/R and were treated with PDRN or Se alone or in association for 30 days. I/R significantly increased hypoxia-inducible factor 1-α (HIF-1α) in Leydig cells, malondialdehyde (MDA), phosphorylated extracellular signal-regulated kinases 1/2 (pErk 1/2), and apoptosis decreased testis weight, glutathione (GSH), testosterone, nuclear factor erythroid 2-related factor 2 (Nrf2), induced testicular structural changes, and eliminated HIF-1α spermatozoa positivity. The treatment with either PDRN or Se significantly decreased MDA, apoptosis, and HIF-1α positivity of Leydig cells, increased testis weight, GSH, testosterone, and Nrf2, and improved the structural organization of the testes. PDRN and Se association showed a higher protective effect on all biochemical, structural, and immunohistochemical parameters. Our data suggest that HIF-1α could play important roles in late testis I/R and that this transcriptional factor could be modulated by PDRN and Se association, which, together with surgery, could be considered a tool to improve varicocele-induced damages.

## 1. Introduction

Testis torsion is one of the most common testicular lesions in the pediatric population [1], with an estimated occurrence of 3.5 per 100,000 persons per year [2]. It causes severe structural damage to the testes, thus leading to infertility [2]; therefore, it must be treated promptly to avoid testicular dysfunction [3]. Events occurring during testicular torsion and subsequent detorsion basically depend on the occurrence of ischemic events and their extension [4] and are comparable to the ischemia-reperfusion (I/R) injury also observed in other organs [5]. 

Experimentally, testis I/R can be obtained with different procedures. In fact, it can be induced by torsion of the spermatic cord, followed by detorsion, both procedures lasting different lengths of time [6,7]. Another procedure is the use of a small microvascular clump placed on the spermatic cord in order to block the spermatic vessels; then, the clump is removed, allowing the reperfusion of the testis [8,9]. Under these circumstances, testes are damaged as a direct consequence of the increased production of reactive oxygen species (ROS) responsible for oxidative stress, and of proinflammatory cytokines and transcription factors, changes in adhesion molecules, enhanced apoptosis, and hormonal unbalance, all leading to germ cell death, aspermatogenesis, and testicular atrophy [10,11].

Even if the testicular environment requires low oxygen tension, an increased concentration of ROS causes peroxidation of lipids present in the sperm plasma membrane, leading to the formation of many toxic by-products, such as malondialdehyde (MDA), able to change the morphological and functional characteristics of cellular membranes [12]. As a result, seminiferous epithelium undergoes irreversible damage [7]. As to the proinflammatory cytokines, they are physiologically produced in the testis [13], being involved in the assemblage or disruption of the blood–testis barrier (BTB) [14]. However, the failure in this steady environment typical of I/R could increase their production, in particular of tumor necrosis factor (TNF)-α, interleukin (IL)-1α, and IL-1β [15,16,17]. The increased levels of these cytokines are responsible for damage in spermatogenesis, causing claudin-11 deregulation [18]. I/R is also able to trigger the apoptosis cascade, inducing further degeneration of the germinal epithelium [19]. Even if apoptosis normally occurs in mammal spermatogenesis to warrant cell homeostasis [20], in I/R-challenged animals, an increase in the number of TUNEL-positive cells and the expression of Bax, caspase-1, and -3 were observed [15,21]. In experimental testis I/R, changes in the hormonal balance involved in male gonad activity were observed. In fact, decreased serum testosterone, FSH, LH, and inhibin levels were described [22,23], indicating a negative effect in controlling testicular functions. I/R injury is also accompanied by changes in hypoxia-inducible factor-1α (HIF-1α) levels. This transcription factor is present in both normoxic and ischemic testes [24], but an evident increase in its expression in I/R-treated rats was demonstrated [16,17], thus indicating a strong link between HIF-1α and I/R injury. However, its role has not yet been completely elucidated. 

In order to counteract I/R effects, numerous therapeutic approaches acting on the different pathways have been tried, either with natural substances [25,26,27] or with nutraceuticals [28,29,30]. Among them, selenium (Se), a trace element able to lower oxidative stress [31,32], reduced MDA levels and increased superoxide dismutase (SOD) activity and ameliorated structural damages after testicular experimental I/R [33]. Kara et al. [34] showed a protective effect of Se administration in I/R testis as indicated by lower lipid peroxidation and apoptosis and by mitigated histopathological damages, thus suggesting an anti-inflammatory and antiapoptotic role of this element in improving fertility. Polydeoxyribonucleotide (PDRN) is an agonist of the A2A adenosine receptor, whose role in an experimental model of I/R testicular injury was evaluated [6]. Its administration ameliorated the histological damages and increased vascular endothelial growth factor (VEGF)-mRNA and VEGF expression in both operated and contralateral testes after 30 days of reperfusion. Additionally, it significantly increased VEGF-receptor 1 expression in Leydig cells. It was suggested that the PDRN plays a role in inducing the synthesis of VEGF, able to ameliorate local perfusion in testis [35].

In light of this background, we investigated I/R injury in a long-term experimental model of testis torsion and detorsion to better understand the behavior of HIF-1α and the possible protective role of Se and PDRN.

## 2. Results

### 2.1. Effects of PDRN, Se, and Their Association on Testis Weight 

The weight of both testes of all the examined groups is shown in Table 1. No significant differences were observed in all sham groups; therefore, for the sake of simplification, a single value is provided as representative of the sham. I/R rats showed operated and contralateral testes weight significantly lower than a sham, even if the contralateral testis weight was greater than operated. In both I/R rats treated with PDRN or Se alone, both operated and contralateral testes weight was significantly lower than sham rats. In I/R rats treated with PDRN plus Se, the weight of both operated and contralateral testes was improved and close to sham.

### 2.2. Effects of PDRN, Se, and Their Association on Testosterone Levels

The levels of testosterone in all the examined groups are shown in Table 1. Testosterone levels were normal in all sham groups; therefore, for the sake of simplification, a single value is provided as representative of the sham. A sharp, significant decrease was observed in I/R animals when compared with sham (−56%). PDRN or Se alone administration caused an increase in testosterone levels when compared with I/R rats (+39% and +41%, respectively), even if significantly lower than sham groups. Only in I/R + PDRN + Se rats was testosterone level close to sham (+53% compared with I/R rats).

### 2.3. Effects of PDRN, Se, and Their Association on Oxidative Stress Parameters

In all sham groups, GSH levels showed basal values; therefore, for the sake of simplification, a single value is provided as representative of the sham. In the I/R group, a significant decrease was observed in both testes versus sham. In both I/R plus PDRN and I/R plus Se groups, the treatment significantly increased GSH levels only in contralateral testes. Only in I/R + PDRN + Se rats the GSH levels were close to normal in both operated and contralateral testes (Table 1). 

In all sham groups, MDA levels showed basal values; therefore, for the sake of simplification, a single value is provided as representative of the sham. In contrast, in I/R animals, MDA levels were significantly increased. The treatment with PDRN or Se alone decreased MDA levels in operated testes, even if not significantly, while the decrease was significant in contralateral testes. Only in I/R + PDRN + Se rats were MDA levels close to normal in both operated and contralateral testes (Table 1).

### 2.4. Effects of PDRN, Se, and Their Association on Nrf2 and pErk 1/2 Expression

**Figure 1:**
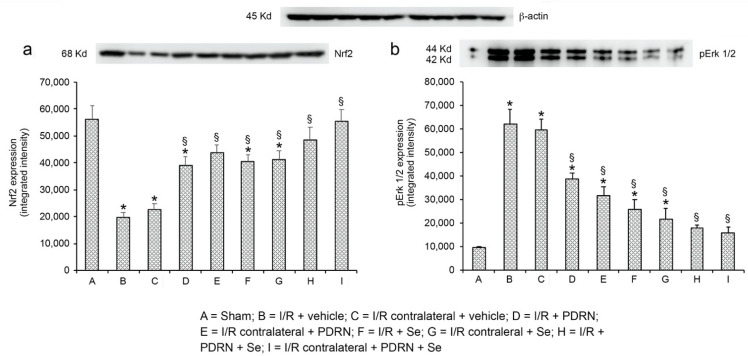
Representative Western blot analysis of Nrf2 (**a**) and pErk 1/2 (**b**) in testes of sham, I/R + vehicle, I/R contralateral + vehicle, I/R + PDRN, I/R contralateral + PDRN, I/R + Se, I/R contralateral + Se; I/R + Se + PDRN; I/R contralateral + Se + PDRN rats. * *p* ≤ 0.05 versus sham; § *p* ≤ 0.05 versus I/R. Bars represent the mean ± SD of seven experiments.

As to Nrf2 expression (Figure 1a), an evident expression was demonstrated in sham animals, with no differences among groups: therefore, for the sake of simplification, a single value is provided as representative of the sham. In contrast, a significant, dramatic decrease (−65% and −60%, respectively) of Nrf2 expression was observed in operated and contralateral testes of I/R rats. In I/R + PDRN group, Nrf2 expression significantly increased versus the I/R group in both operated and contralateral testes (+50% and +52%, respectively). Similarly, an increase in Nrf2 expression was observed in I/R + Se group, higher in operated testes (+51%) and lower in contralateral testes (+45%). In I/R + PDRN + Se rats, Nrf2 expression was comparable to sham.

When pErk 1/2 expression was evaluated (Figure 1b), its intensity was low and superimposable in all sham groups; therefore, for the sake of simplification, a single value is provided as representative of the sham. In both operated and contralateral testes of the I/R group, pErk 1/2 expression was significantly increased (+85% and +84 % versus sham, respectively). In I/R + PDRN group, a significant decrease of pErk 1/2 expression was observed (−38% and −47% versus the I/R group, respectively). In the testes of I/R + Se rats, a further decrease of pErk 1/2 expression was found in the left and the right testes (−49% and −64%, respectively) versus the corresponding I/R testes. In I/R + PDRN + Se rats, pErk 1/2 expression was similar to sham.

### 2.5. Effects of PDRN, Se, and Their Association on Testis Structure

**Figure 2:**
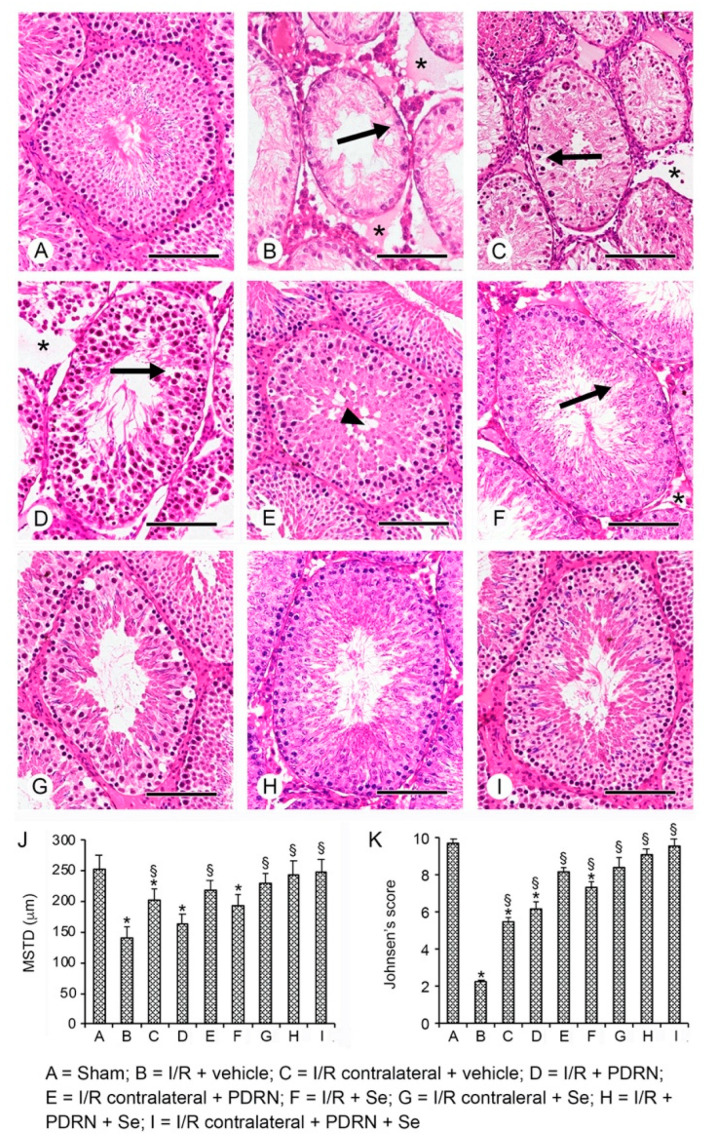
Histological findings in the seminiferous tubules of the different groups of rats (H&E stain). (**A**) Sham rats show normal morphology. (**B**) In I/R + vehicle rats, degenerative changes in the germinal cells (arrow) and edema of the extratubular compartment (*) are evident. (**C**) CL testes of I/R + vehicle rats show a germinal epithelium with only spermatogonia, isolated spermatocytes (arrow), and interstitial edema (*). (**D**) In the testes of I/R + PDRN rats, elongated spermatids are present in the germinal epithelium, even if some intercellular clefts (arrow) are evident; mild edema is demonstrated in the extratubular compartment (*). (**E**) The CL testes of the same group show a close-to-normal extratubular compartment and a germinal epithelium with isolated, empty zones in the basal position. (**F**) The testes of I/R + Se rats have tubules with peripheral spermatogonia and detached spermatocytes; the extratubular compartment is dilated (*). (**G**) CL testes of the same group show larger tubules, and their lumen is filled with round spermatids (arrowhead). (**H**,**I**) In the testes of I/R + PDRN + Se rats and the CL testes of the same group, no evident morphological changes are recognized in both the tubular and the extratubular compartments. (**J**) Histogram of the mean seminiferous tubule diameter (MSTD) in the different groups of rats (mean ± standard deviation). (**K**) Histogram of the Johnsen score in the different groups of rats (mean ± standard deviation). * *p* ≤ 0.05 versus sham group; § *p* ≤ 0.05 versus I/R rats (Scale bar: 100 µm).

All the sham groups of rats showed seminiferous tubules and extratubular compartments with normal morphology; therefore, for the clarity of images, a single micrograph is provided as characteristic of all shams (Figure 2A,J,K). After 30 days of reperfusion, the operated I/R group testes showed evident tubular changes with few spermatogonia and much cellular debris, significantly reduced MSTD, and a very low Johnsen score. In the extratubular compartment, evident interstitial edema was present (Figure 2B,J,K). In CL testes of the same group, the germinal epithelium was formed by some spermatogonia and isolated spermatocytes; the extratubular compartment was edematous (Figure 2C,J,K). In I/R + PDRN alone-treated rats, the germinal epithelium was better preserved with elongated spermatids, even if some intercellular clefts were present; in the extratubular compartment, interstitial edema was still evident (Figure 2D,J,K). CL testes of the same group showed a germinal epithelium with isolated, empty zones in the basal position and a close-to-normal extratubular compartment. MSTD and Johnsen scores were significantly improved (Figure 2E,J,K). In I/R + Se alone-treated rats, tubules with peripheral spermatogonia and few detached spermatocytes were present; MSTD and Johnsen scores were significantly higher compared with the I/R group but lower compared with I/R plus PDRN alone group; the extratubular compartment was dilated (Figure 2F,J,K). CL testes of the same group had larger tubules, as indicated by MSTD, and round spermatids in their lumen (Figure 2G,J,K). In I/R rats treated with the association Se and PDRN, only mild edema of the extratubular compartment was present (Figure 2H,J,K), while CL testes of the same animal group showed a normal organization (Figure 2I–K).

### 2.6. Effects of PDRN, Se, and Their Association on Apoptosis with TUNEL Assay

**Figure 3:**
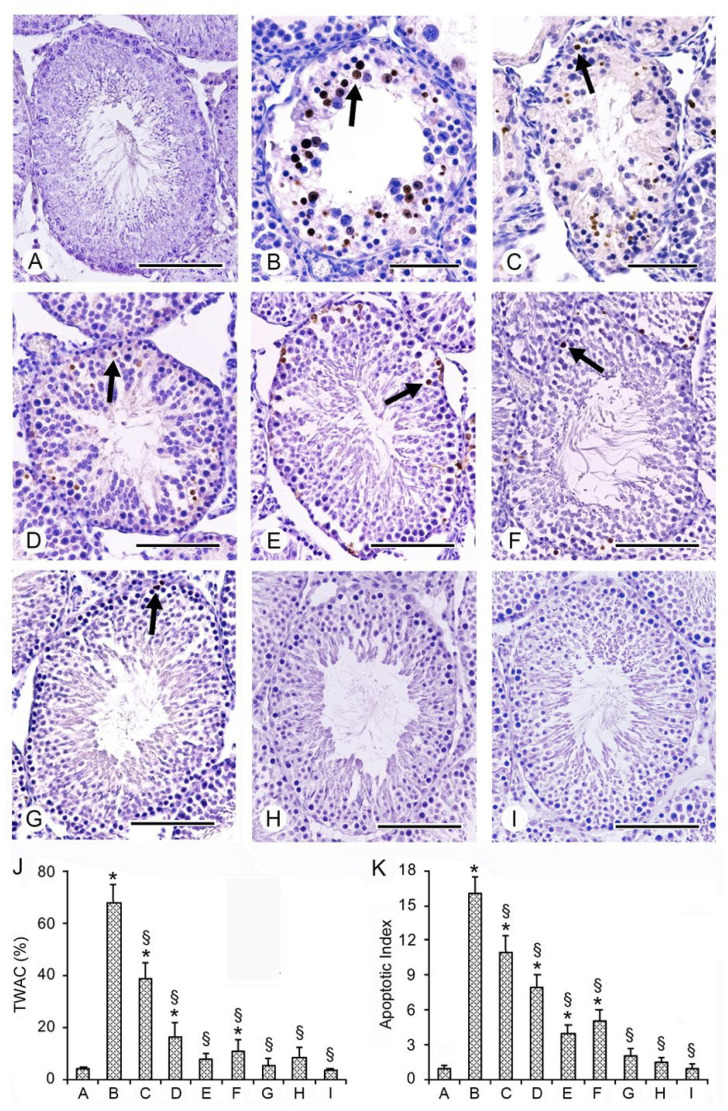
Assessment of apoptosis in the different groups of rat testes with TUNEL stain. (**A**) In the testes of all sham rats, no TUNEL-positive cells were present. (**B**,**C**) Many TUNEL-positive cells (arrow) were seen in the wall of the seminiferous tubules of I/R + vehicle rats and in CL testes of the same group. (**D**,**E**) In I/R + PDRN alone-treated rats and in the CL testes of the same group, TUNEL-positive cells (arrow) were decreased when compared with I/R rats. (**F**,**G**) In I/R + Se-treated rats and in CL testes of the same group, only isolated TUNEL-positive cells were present. (**H**,**I**) In I/R rats treated with the association PDRN + Se, in both operated and CL testes, only rarely isolated TUNEL-positive germ cells were observed. (**J**) Tubules with apoptotic cells (TWAC) (expressed in %) in the different groups of rats. (**K**) Apoptotic index values (apoptotic cells/tubule) in the different groups of rats. * *p* ≤ 0.05 versus sham group; § *p* ≤ 0.05 versus I/R rats (scale bar: (**A**,**D**–**I**) = 100 μm; (**B**,**C**) = 50 μm).

In the seminiferous tubules of all sham rats, no TUNEL-positive cells were present; therefore, for the clarity of images, a single micrograph is provided as characteristic of all groups (Figure 3A,J,K). In contrast, in the wall of the seminiferous tubules of I/R and CL testes of the same group, an evident positivity was observed (Figure 3B,C). In fact, both TWAC and apoptotic index were significantly higher when compared with the sham group (Figure 3J,K). In I/R rats treated with PDRN alone, an evident decrease in TUNEL-positive cell number in TWAC and in the apoptotic index was observed (Figure 3D,J,K) compared with I/R rats. CL testes of the same group showed a close to normal TWAC, while the apoptotic index was still significantly higher (Figure 3E,J,K). In I/R and CL testes of rats treated with Se alone, only isolated TUNEL-positive spermatogonia were present (Figure 3F,G); TWAC and apoptotic index values were significantly reduced (Figure 3J,K). In I/R rats treated with Se + PDRN, both operated and CL testes showed isolated TUNEL-positive germ cells in the periphery of the seminiferous tubules (Figure 3H,I); TWAC and apoptotic index were close to sham (Figure 3J,K).

### 2.7. Effects of PDRN, Se, and Their Association on HIF-1α Activity

**Figure 4:**
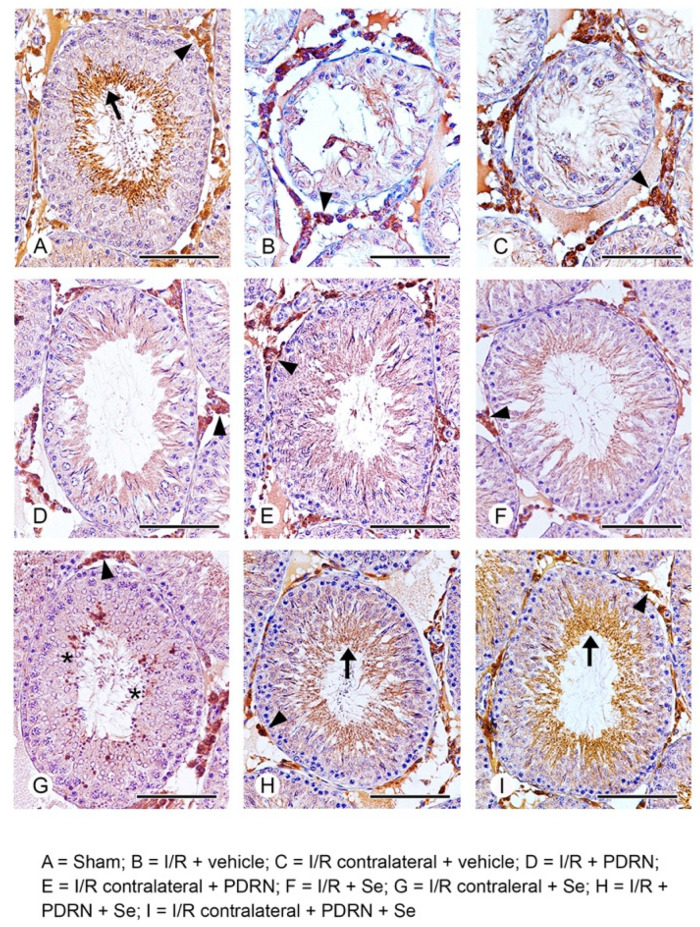
Immunohistochemical expression of HIF-1α in the testes. (**A**) In sham groups, HIF-1α positive elongated spermatids and spermatozoa (arrow) and Leydig cells (arrowhead) are present. (**B**,**C**) In both operated and CL testes of I/R + vehicle rats, no HIF-1α positive cells are evident in the highly damaged tubular wall; interstitial Leydig cells (arrowhead) show a higher expression versus sham rats. (**D**) In the operated testes of I/R + PDRN-treated rats, a moderate positivity for HIF-1α of the tails of spermatozoa (arrow) and Leydig cells (arrowhead) is evident. (**E**) In CL testes of I/R + PDRN-treated rats, isolated HIF-1α positive spermatocytes and spermatids (asterisk) are present in the seminiferous epithelium, in addition to Leydig cells (arrowhead). (**F**,**G**) In both operated and CL testes of I/R + Se-treated rats, no HIF-1α positive cells are present in the otherwise improved structure of seminiferous tubules; the expression of Leydig cells (arrowhead) is reduced when compared with I/R rats. (**H**,**I**) In the seminiferous tubules of both operated and CL testes of I/R + PDRN + Se-treated rats, an evident positivity for HIF-1α of the tails of spermatozoa (arrow) and Leydig cells (arrowhead) similar to sham is present. (Scale bar: (**A**,**D–I**) = 100 µm; (**B**,**C**) = 50 µm).

In the seminiferous tubules of all sham groups, HIF-1α positive elongated spermatids and spermatozoa were present, while in the interstitial spaces, HIF-1α positive Leydig cells were observed; therefore, for the clarity of images, a single micrograph is provided as characteristic of all shams (Figure 4A). In both operated and CL testes of I/R rats, no HIF-1α positive cells were demonstrated in the highly damaged wall of the tubules, while Leydig cells showed a higher expression when compared with sham rats (Figure 4B,C). In the operated testes of I/R + PDRN-treated rats, a moderate positivity for HIF-1α of the tails of spermatozoa and Leydig cells was present (Figure 4D), while in CL testes of the same group, isolated HIF-1α positive spermatocytes and spermatids were demonstrated, in addition to Leydig cells (Figure 4E). In both operated and CL testes of I/R + Se-treated rats, no HIF-1α positive cell was present in the otherwise improved structure of seminiferous tubules; in the interstitial spaces, the expression of Leydig cells was mildly reduced when compared with I/R rats (Figure 4F,G). In the seminiferous tubules of both operated and CL testes of I/R rats treated with Se + PDRN, diffuse positivity for HIF-1α of spermatozoa and HIF-1α positive Leydig cells similar to sham was demonstrated (Figure 4H,I).

## 3. Discussion

Among pediatric emergencies, the torsion of the spermatic cord needs immediate surgical treatment to restore the testicular vascular supply; if not treated, testis damages occur, leading to infertility [36]. However, the reperfusion of ischemic tissues is responsible for further structural and functional damage of all components of the gonad; in fact, it was considered superimposable to the I/R damages demonstrated in other organs, such as heart, kidney, liver, and gastrointestinal apparatus [37,38].

Many studies examining the effects of different substances in counteracting the different mechanisms involved in testicular I/R damage were performed, testing the role of vitamin C [39], the melanocortin analog [Nle(4),D-Phe(7)]α-melanocyte-stimulating hormone [40], α-lipoic acid [41], ghrelin [42], melatonin [43], astaxanthin [7], and relaxin [44].

Additionally, Se, a trace element, was able to reduce oxidative stress [31,32], apoptosis [34], and to improve structural damages [33] after experimental I/R in testis, thus suggesting an anti-inflammatory and antiapoptotic role of this element in improving fertility.

The role of PDRN, an agonist of the A2A adenosine receptor, in an experimental model of I/R testicular injury was evaluated, and it was suggested that it induced the synthesis of VEGF [6], able to ameliorate local perfusion in testis [35].

It has been shown that the original factor in male infertility is oxidative damage. In the body, cells produce ROS during their normal metabolic activity, and there is an increasing evidence that they are beneficial even under certain pathological conditions [45]. In the testis, MDA as a lipid peroxidation marker and GSH as an antioxidant, are able to modulate oxidative stress, but I/R injury causes excessive production of ROS [46]. Our data indicate that both PDRN and Se, alone and in association, were able to interact significantly with the oxidative stress induced by experimental I/R, normalizing MDA and GSH levels in testes. Indeed, Se has a direct well-defined antioxidant activity through different pathways due to its incorporation in selenoproteins, which are involved in the regulation of antioxidant activities. In particular, in the testis Se-dependent glutathione peroxidase detoxifies cellular peroxides that protect against ROS [47,48]. On the contrary, PDRN could have an indirect effect on the oxidative stress induced by ROS. Probably, the antioxidant activity of PDRN could be related either to its effect on inflammation and its related oxidative stress, or could be due to a synergic effect with Se [6,49]. Accordingly, our present experimental data suggest that the co-administration of PDRN and Se, further improved the overall antioxidant activity of treatment in comparison with single administration of both molecules.

In addition, the amplified production of ROS in testis I/R stimulates the MAPK family and triggers the inflammatory cascade and the apoptosis machinery. In fact, increased concentrations of TNF-α and IL-1β were demonstrated [50], thus confirming a negative function of inflammation during testicular I/R. In our study, we demonstrated an evident increase in pErk 1/2 levels, whose role in the molecular mechanism of the inflammatory response has been assessed [51]. Both PDRN and Se, alone and in association, significantly reduced pErk 1/2 levels, indicating a positive role in reducing inflammation in I/R testes.

Furthermore, I/R triggers different programs of cell death, such as necrosis and apoptosis. While necrosis induces swelling and breakup of cells and organelles, stimulating the inflammatory process and cytokine production, apoptosis is characterized by shrinkage of cells and nuclei but by plasma membrane integrity [52]. In I/R rats, a large number of TUNEL-positive germ cells was demonstrated mainly in the basal layer of the seminiferous tubules, suggesting that apoptosis is highly increased in operated and contralateral testes. From our data, it was possible to show that both PDRN and Se, alone and in association, were able to improve significantly apoptosis induced by experimental I/R.

Lastly, we confirmed that I/R rats showed dramatic changes in the seminiferous epithelium and a very low Johnsen score. The administration of PDRN and Se, alone or in association, significantly ameliorated spermatogenesis, as demonstrated by histological micrographs, improved Johnsen score, and MSTD.

Particular attention was paid to the behavior of HIF-1α in rats with I/R testicular injury treated with PDRN and Se, alone or in association. 

The increased expression of HIF-1α observed in I/R rats could be related to the corresponding elevated levels of pErk 1/2 observed with WB analysis. In fact, an interaction between HIF-1α and pErk 1/2 was demonstrated [53], indicating cooperation between hypoxic and growth factor signals that finally led to the surge in HIF-1-mediated gene expression.

As to the tubular compartment, the seminiferous epithelium of sham rats showed an evident HIF-1α expression of elongated spermatids and spermatozoa. According to the current literature, HIF-1α positivity in the abluminal compartment of the seminiferous tubules was present in the flagellum of spermatozoa of mice testes under normoxic conditions [54] and in elongated spermatids of adult mice [55]. It was suggested that HIF-1α might play a protective role for haploid male germ cells located in the luminal part of the seminiferous tubules. In these regions, owing to the greater distance from blood vessels, O_2_ tension is lower than that of the interstitial region or basal regions, where spermatogonia undergo a self-renewal process [56]. Therefore, the luminal regions are particularly hypoxic, and the basal expression of HIF-1α indicates a response to the lower access to O_2_ [54]. The other cells of the seminiferous epithelium were instead HIF-1α negative, differently from the data of Takahashi et al. [55] obtained from mice testes. In I/R rats, owing to the dramatic structural changes of the seminiferous tubules induced by the experimental procedure [16,17], HIF-1α positivity was absent. The treatment with Se and PDRN, alone or in association, was able to restore the structural organization of the seminiferous tubules so that in I/R rats treated with both PDRN and Se, diffuse positivity for HIF-1α of spermatozoa was demonstrated.

As to the extratubular compartment, we confirmed an evident positivity for HIF-1α of Leydig cells in sham rats [10]. The constitutive expression of HIF-1α, even in normoxic conditions, could be related to the low, similar to hypoxia [10,24], physiological O_2_ tension in the interstitial tissue of the rat testis, compared with other organs [50]. However, in our study, differently from previous data and probably related to the time of reperfusion [10], I/R rats HIF-1α showed an increased expression in Leydig cells. The basal positivity to HIF-1α and its increase after 30 days of I/R can induce another important effect in Leydig cells.

The latter could be related to the absence of apoptosis in these cells [57], related to the expression, even in normoxic conditions, of HIF-1α [10]. In fact, it was proposed that HIF-1, in particular HIF-1α as the predominant HIF-1 expressed in the rat testis [10], may induce the activation of antiapoptotic genes able to shield Leydig cells from apoptosis either in sham or in I/R testes.

Another important consequence of the increased expression of HIF-1α in I/R rats is the decrease in testosterone levels. Testosterone reduction in I/R animals was previously related to oxidative stress, able to inhibit androgenesis in Leydig cells [23,58]. However, a key role of HIF-1 in testosterone synthesis was recently demonstrated under hypoxia conditions. In fact, after 28-day treatment of intermittent hypoxia, a significant decrease in serum testosterone was observed in mice [59]. Therefore, the increased expression of HIF-1α in Leydig cells during I/R could justify the reduced levels of testosterone in serum observed in our animals, which was significantly reversed after the treatment with PDRN and Se alone or in association, parallel to the reduction in HIF-1α expression.

This finding is of particular interest; in fact, it is possible to speculate that the HIF-1α pathway could represent another mechanism for the development of late complications related to testicular torsion. However, further studies are necessary to better understand the role of this transcriptional factor in testis I/R. 

## 4. Materials and Methods

### 4.1. Ethical Approval

The standards for the care and use of animal subjects, as stated in the ARRIVE (Animal Research: Reporting In Vivo Experiments) guidelines [60], were followed in the present work. All procedures were approved by the Italian Ministry of Health (authorization number 112/2017-PR) and by the Institutional Animal Care and Use Committee of the University Hospital of Messina, Messina, Italy.

### 4.2. Experimental Protocol

Male Sprague Dawley rats (250–300 g) were housed and maintained under specific pathogen-free conditions at the animal facility of the School of Medicine at the University of Messina, Messina, Italy. Rats were provided a standard diet ad libitum with free access to tap water and were maintained on a 12-h light/dark cycle. The animals (total number = 56) were divided into 8 groups of 7 animals each. Four groups (sham rats) were anesthetized and operated on, but no testicular torsion and consequent ischemia were performed. These animals were divided into the following groups: one group treated with vehicle (0.9% NaCl), one group treated daily with PDRN (8 mg/kg) alone, one group treated daily with Se (3 mg/kg) alone, and one group treated daily with both Se (3 mg/kg) and PDRN (8 mg/kg). The animals of the other four groups were anesthetized, and the torsion of the left testis and spermatic cord was performed for 1 h, as previously described [61]. Then the testis was detorted. These animals were divided into the following groups, all treated for 30 days after the detorsion: one group (I/R + vehicle), one group (I/R + PDRN) treated daily with PDRN (8 mg/kg) alone, one group (I/R + Se) treated daily with Se (3 mg/kg) alone, and one group (I/R + Se + PDRN) treated daily both Se (3 mg/kg) and PDRN (8 mg/kg). The doses of both PDRN and Se were selected on the basis of the results of previous works [52,62]. The animals of each group were sacrificed after 30 days from the surgical treatment with an overdose of ketamine and xylazine, and bilateral orchidectomies were performed. The limit of 30 days of administration of PDRN and selenium was chosen on the basis of the previous works which showed that at that time structural and functional damages were particularly evident and typical of detorsion [6,40,42,63,64]. The testes were collected, weighed, and treated for the following experimental procedures.

### 4.3. Isolation of Soluble Proteins 

Isolation of soluble proteins from testis samples (about 30 mg) of the different groups was performed in 1 mL of lysis buffer [25 mM Tris/HCL, pH 7.4, 1.0 mM EGTA, 1.0 mM EDTA, 0.5 mM phenyl methylsulfonyl fluoride, aprotinin, leupeptin, pepstatin A (10 ug/mL each) and Na_3_VO_4_ 100 mM] and homogenized with a Dounce homogenizer [65]. The homogenate was centrifuged at 15,000 g for 15 min, and the supernatant was collected and used for total protein determination with the Bio-Rad protein assay kit (Bio-Rad, Richmond, CA, USA).

### 4.4. Determination of GSH and MDA Levels

GSH level was determined in the testes of all groups using Ellman’s reagent, and the results were expressed as µmol/g tissue [66]. MDA level was obtained from products of lipid peroxidation, and the results were expressed as nmol/g tissue [66].

### 4.5. Determination of Testosterone Levels

An ELISA kit was used for testosterone levels in serum, according to the protocol suggested by the manufacturer. In brief, blood was obtained from cardiac puncture, and serum was achieved by centrifugation for 10 min at 1000 g. An HRP conjugate and the specific antibody were added, followed by substrates and a stop solution. The mean absorbance was calculated using a microplate reader at 450 nm and correlated with those from standard curves. Data were expressed in ng/mL.

### 4.6. Determination of Nrf2 and pErk 1/2 by Western Blot Analysis

Total proteins (30 µg) were denatured with reducing buffer [62 mMTris (pH 6.8), 10% glycerol, 2% sodium dodecyl sulfate, 5% β-mercaptoethanol, and 0.003% bromophenol blue], separated by electrophoresis and then transferred onto a polyvinylidene difluoride (PVDF) membrane using a transfer buffer [39 mM glycine, 48 mM Tris (pH 8.3)] at 200 mA for 1 h. In order to block membrane proteins, 5% nonfat dry milk in TBS-0.1% Tween for 1 h at room temperature was used, followed by three washes with TBS-0.1% Tween for 10 min each, then incubated with a primary antibody for Nrf2 (Abcam, Cambridge, UK) and pErk 1/2 (Cell Signaling, Beverly, MA, USA), diluted 1:1000 in TBS-0.1% Tween overnight at 4 °C. The membranes were washed three times for 10 min each with TBS-0.1% Tween to eliminate nonspecific bindings and incubated with a specific peroxidase-conjugated secondary antibody (Genetex, Irvine, CA, USA), diluted 1:5000, for 1 h at room temperature. After three washes, the membranes were analyzed by the enhanced chemiluminescence system according to the manufacturer’s protocol (Amersham Biosciences, Amersham, UK). Equal loading of proteins was assessed on stripped blots by immunodetection of β-actin with a rabbit monoclonal antibody (Cell Signaling, Beverly, MA, USA) diluted 1:1000 and peroxidase-conjugated antirabbit IgG (Genetex, Irvine, CA, USA) diluted 1:5000. The results from each experimental group were obtained from 7 samples of each experimental group. The results were normalized versus β-actin and expressed as relative integrated intensity compared with those of sham rats measured with the same batch. All the target proteins were run on the same membrane after stripping the primary antibody with a glycine solution. β-actin was used as a control on the same stripped blot.

### 4.7. Histological Evaluation

The testes were fixed in Bouin, dehydrated in ethanol, cleared in xylene, and embedded in paraffin (Paraplast, SPI Supplies, West Chester, PA, USA). Sections of 5 µm thickness were mounted on glass silanized slides and stained with hematoxylin and eosin (H&E). Photomicrographs were taken with a Nikon Ci-L (Nikon Instruments, Tokyo, Japan) light microscope, using a digital camera Nikon DS-Ri2. Images, saved as Tagged Image Format Files (TIFF), were printed at the same final magnification and blindly assessed by two trained observers without knowledge of the previous treatment. Five microscopic fields, all including two entire seminiferous tubules from ten not serial sections of each group, were considered. 

The mean seminiferous tubule diameter (MSTD) was calculated by measuring the diameters of 100 separate seminiferous tubules, all showing a circular profile. A Peak Scale Loupe 7× (GWJ Company, Hacienda Heights, CA, USA) micrometer was used as a scale calibration standard to calculate the mean diameters expressed in micrometers (µm). Furthermore, from the same tubules, the germinal epithelium was evaluated using the Johnsen scoring system [67], as modified by Erdemir et al. [68]. In brief, each tubule was scored from 10 to 1 according to the germinal epithelium organization: 10 = whole spermatogenesis and normal tubules; 9 = many spermatozoa and disorganized spermatogenesis; 8 = only a few spermatozoa; 7 = no spermatozoa but many spermatids; 6 = only a few spermatids; 5 = no spermatozoa or spermatids but many spermatocytes; 4 = only a few spermatocytes; 3 = only spermatogonia; 2 = no germ cells but only Sertoli cells; 1 = no germ cells and no Sertoli cells. 

### 4.8. Terminal Deoxynucleotidyl Transferase dUTP Nick End Labeling (TUNEL) Assay for Apoptosis

A kit for the assay of apoptosis (In situ Apoptosis Detection kit, Abcam, Cambridge, UK) was used, and 5 µm sections were obtained from the same samples used for morphological evaluation. After clearing in xylene and rehydration in ethanol, permeabilization with proteinase K was performed, followed by endogenous peroxidase blocking with 3% H_2_O_2_ in methanol. Sections were treated with terminal deoxynucleotidyl transferase, biotin-labeled deoxynucleotides, streptavidin–horseradish peroxidase conjugate, and then with diaminobenzidine as chromogen. Micrographs were taken with a Nikon Ci-L light microscope using a digital camera Nikon Ds-Ri2. One hundred seminiferous tubules per group were blindly evaluated by two trained observers to determine the percentage of tubules with apoptotic cells (%TWAC) and the mean number of TUNEL-positive cells per tubule (apoptotic index) [55].

### 4.9. Immunohistochemistry for HIF-1α

From the same blocks used for histological evaluation, paraffin-embedded 5 µm sections were mounted on Polysine slides (Thermo Fisher Scientific, Waltham, MA, USA), cleared in xylene, and rehydrated in ethanol. Antigen retrieval was performed with pH 6.0 buffer citrate and endogenous peroxidase blocking with 0.3% H_2_O_2_ in methanol. The primary antibody (HIF-1α, 1:100, Orizor Scientific, Messina, Italy) was incubated overnight at 4 °C in a moisturized chamber, and the day after, the secondary antibody (Pierce anti-mouse, Cambridge, UK) was added. The reaction was visualized with 3,3′-Diaminobenzidine (DAB) (Sigma-Aldrich, Milan, Italy). Counterstaining was performed in Mayer’s haematoxylin. Slides were photographed with a Nikon Ci-L (Nikon Instruments, Tokyo, Japan) light microscope.

### 4.10. Drugs

Mastelli srl, Sanremo, Italy, kindly provided PDRN. PDRN is extracted and purified at high temperature, so to regain a >95% pure active constituent with inactivated proteins and peptides. It contains polydeoxyribonucleotide, NaCl and water. Se was used as a powder, purity 99.99% with <150.0 ppm metal traces, −100 mesh and was purchased from Sigma Aldrich, Milan, Italy. All the chemicals and reagents were commercially available reagent grades. 

### 4.11. Statistical Analysis

The different experimental groups were analyzed by the Student’s *t*-test and one-way ANOVA with Tukey’s post-test for intergroup comparisons. Values are expressed as mean ± standard deviation (SD). A *p* value of ≤0.05 was considered statistically significant.

## Figures and Tables

**Table 1:** Effects on testis weight, testosterone, glutathione, and malondialdehyde levels induced by PDRN and Se alone or in association in testes of I/R rats compared with ischemia-reperfusion and sham rats. All values are expressed as mean ± SD; n = 7 animals/group.

Groups	Testis Weight (g)	Testosterone (ng/mL)	GSH (µmol/g Tissue)	MDA (nmol/g Tissue)
**Sham**	1.71 ± 0.21	5.7 ± 0.4	344 ± 14	5.4 ± 0.3
**I/R + vehicle**	0.93 ± 0.32 ^a^	2.5 ± 0.4 ^a^	262 ± 15 ^a^	11.8 ± 0.4 ^a^
**I/R CL + vehicle**	1.38 ± 0.35 ^a^		271 ± 13 ^a^	9.8 ± 0.3 ^a^
**I/R + PDRN**	1.40 ± 0.29 ^a,b^	4.1 ± 0.3 ^a,b^	294 ± 16 ^a^	8.7 ± 0.5 ^a^
**I/R CL + PDRN**	1.55 ± 0.27 ^a,b^		305 ± 14 ^a,b^	7.2 ± 0.3 ^a,b^
**I/R + Se**	1.44 ± 0.33 ^a,b^	4.4 ± 0.5 ^a,b^	303 ± 13 ^a,b^	8.1 ± 0.5 ^a,b^
**I/R CL + Se**	1.59 ± 0.25 ^a,b^		316 ± 15 ^a,b^	6.8 ± 0.4 ^a,b^
**I/R + PDRN + Se**	1.62 ± 0.27 ^b^	5.3 ± 0.4 ^b^	331 ± 15 ^b^	5.9 ± 0.2 ^b^
**I/R CL + PDRN + Se**	1.70 ± 0.26 ^b^		337 ± 13 ^b^	5.5 ± 0.2 ^b^

a = *p* < 0.05 versus sham; b = *p* < 0.05 versus I/R + vehicle. I/R—ischemia-reperfusion; PDRN—polydeoxyribonucleotide; Se—selenium; GSH—glutathione; MDA—malondialdehyde CL—contralateral testis.

## Data Availability

The data presented in this study are available on request to the corresponding author.
