# Correction: Antonuccio et al. Involvement of Hypoxia-Inducible Factor 1-α in Experimental Testicular Ischemia and Reperfusion: Effects of Polydeoxyribonucleotide and Selenium. *Int. J. Mol. Sci.* 2022, *23*, 13144

**DOI:** 10.3390/ijms26178636

**Published:** 2025-09-05

**Authors:** Pietro Antonuccio, Giovanni Pallio, Herbert Ryan Marini, Natasha Irrera, Carmelo Romeo, Domenico Puzzolo, Jose Freni, Giuseppe Santoro, Igor Pirrotta, Francesco Squadrito, Letteria Minutoli, Antonio Micali

**Affiliations:** 1 Department of Human Adult and Childhood Pathology, University of Messina, 98125 Messina, Italy; pietro.antonuccio@unime.it (P.A.); romeo.carmelo@unime.it (C.R.); amicali@unime.it (A.M.); 2 Department of Clinical and Experimental Medicine, University of Messina, 98125 Messina, Italy; gpallio@unime.it (G.P.); hrmarini@unime.it (H.R.M.); nirrera@unime.it (N.I.); ipirrotta@unime.it (I.P.); fsquadrito@unime.it (F.S.); 3 Department of Biomedical, Dental Sciences and Morphofunctional Imaging, University of Messina, 98125 Messina, Italy; puzzolo@unime.it (D.P.); jose.freni@unime.it (J.F.); giuseppe.santoro@unime.it (G.S.)

In the original publication [1], there was a mistake in Figure 3, Panel A, in which the authors presented a picture very similar to another proposed in a paper published at a different time [2]. The corrected Figure 3 appears below. The authors state that the scientific conclusions are unaffected. This correction was approved by the Academic Editor. The original publication has also been updated.

## Figures and Tables

**Figure 3:**
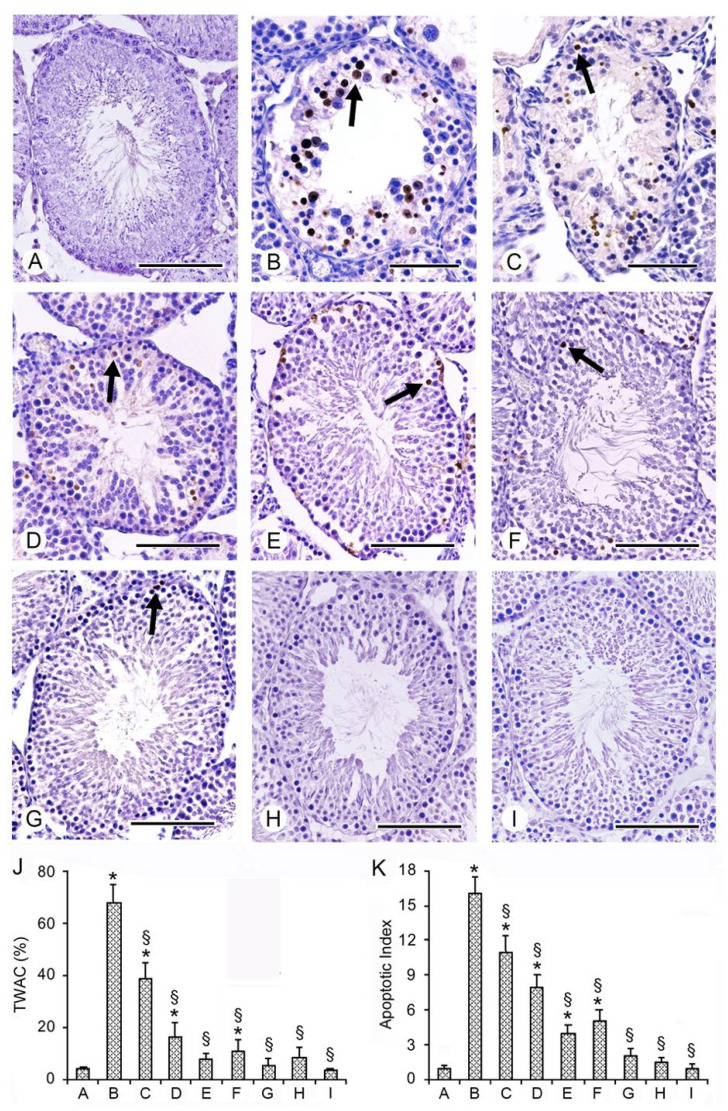
Assessment of apoptosis in the different groups of rat testes with TUNEL stain. (**A**) In the testes of all sham rats, no TUNEL-positive cells were present. (**B**,**C**) Many TUNEL-positive cells (arrow) were seen in the wall of the seminiferous tubules of I/R + vehicle rats and in CL testes of the same group. (**D**,**E**) In I/R + PDRN alone-treated rats and in the CL testes of the same group, TUNEL-positive cells (arrow) were decreased when compared with I/R rats. (**F**,**G**) In I/R + Se-treated rats and in CL testes of the same group, only isolated TUNEL-positive cells were present. (**H**,**I**) In I/R rats treated with the association PDRN + Se, in both operated and CL testes, only rarely isolated TUNEL-positive germ cells were observed. (**J**) Tubules with apoptotic cells (TWAC) (expressed in %) in the different groups of rats. (**K**) Apoptotic index values (apoptotic cells/tubule) in the different groups of rats. * *p* ≤ 0.05 versus sham group; § *p* ≤ 0.05 versus I/R rats (scale bar: (**A**,**D**–**I**) = 100 μm; (**B**,**C**) = 50 μm).
